# Household transmission of COVID‐19 among the earliest cases in Antananarivo, Madagascar

**DOI:** 10.1111/irv.12896

**Published:** 2021-08-10

**Authors:** Rila Ratovoson, Rado Razafimahatratra, Lova Randriamanantsoa, Mihaja Raberahona, Hasina Joelinotahiana Rabarison, Fara Nirintsoa Rahaingovahoaka, Emmanuel Harizaka Andriamasy, Perlinot Herindrainy, Norosoa Razanajatovo, Soa Fy Andriamandimby, Philippe Dussart, Matthieu Schoenhals, Mamy Jean de Dieu Randria, Jean Michel Heraud, Rindra Vatosoa Randremanana

**Affiliations:** ^1^ Institut Pasteur de Madagascar Antananarivo Madagascar; ^2^ Centre Hospitalier Universitaire Anosiala‐Alakamisy Ambohidratrimo‐Antananarivo Madagascar; ^3^ Centre Hospitalier Manara‐Penitra Andohatapenaka Antananarivo Madagascar; ^4^ Centre Hospitalier Universitaire Joseph Raseta Befelatanana Antananarivo Madagascar

**Keywords:** COVID‐19, household, Madagascar, transmission

## Abstract

**Background:**

Households are among the highest risk for the transmission of SARS‐CoV‐2. In sub‐Saharan Africa, very few studies have described household transmission during the COVID‐19 pandemic. Our work aimed to describe the epidemiologic parameters and analyze the secondary attack rate (SAR) in Antananarivo, Madagascar, following the introduction of SARS‐CoV‐2 in the country in March 2020.

**Methods:**

A prospective case‐ascertained study of all identified close contacts of laboratory‐confirmed COVID‐19 infections was conducted in Antananarivo from March to June 2020. Cases and household contacts were followed for 21 days. We estimated epidemic parameters of disease transmission by fitting parametric distributions based on infector‐infected paired data. We assessed factors influencing transmission risk by analyzing the SAR.

**Findings:**

Overall, we included 96 index cases and 179 household contacts. Adjusted with the best‐fit normal distribution, the incubation period was 4.1 days (95% CI 0.7–7.5]). The serial interval was 6.0 days (95% CI [2.4–9.6]) after adjusting with the best‐fit Weibull distribution. On average, each index case infected 1.6 family members (95%CI [0.9–2.3]). The mean SAR among close contacts was 38.8% (95% CI [19.5–58.2]) with the best‐fit gamma distribution. Contacts older than 35 years old were more likely to be infected, and the highest SAR was found among them.

**Conclusion:**

The results of our study provide key insights into the epidemiology of the first wave of SARS‐CoV‐2 in Madagascar. High rates of household transmission were found in Antananarivo, emphasizing the need for preventive measures to reduce community transmission.

## 1 INTRODUCTION

Severe acute respiratory syndrome coronavirus 2 (SARS‐CoV‐2), which is the virus that causes coronavirus disease (COVID‐19), first appeared in Wuhan (Hubei Province), China, in early December 2019. The first cases of pneumonia caused by this virus were reported to the World Health Organization (WHO) on December 31, 2019, where it caused an epidemic that subsequently spread to other countries. The WHO declared COVID‐19 a Public Health Emergency of International Concern on January 30, 2020, and the disease further escalated to the status of a pandemic on March 11, 2020 (1). As a response to the pandemic, governments implemented non‐pharmaceutical interventions (NPIs), including travel bans, border closings, mandatory screening of citizens returning from heavily affected areas, movement restrictions, and partial or complete lockdowns.

Understanding of COVID‐19 comes largely from disease surveillance and epidemiologic studies undertaken in China (2, 3) and high‐income countries (4, 5, 6). However, confirmed cases of COVID‐19 have also occurred in low‐ and middle‐income countries (7, 8). The first confirmed case of COVID‐19 in Africa was reported in Egypt on February 14, followed by Algeria (9). By March, COVID‐19 cases were reported across most of the continent. The first three confirmed COVID‐19 cases were imported to Antananarivo, the capital city of Madagascar, on March 19–20, 2020. Following this notification and with the aim of stopping or slowing down the rate of transmission of SARS‐CoV‐2, the Malagasy government introduced stringent NPIs, such as physical distancing (school closures, work‐from‐home arrangements for civil servants, closing bars and restaurants, and suspension of public leisure). The country exceeded 17 000 confirmed cases and more than 240 total deaths in early November 2020 (10).

During the early phases, testing, contact tracing, quarantine, and isolation were carried out when cases were identified. Uninfected and asymptomatic contacts were often closely tracked, providing information about transmission and natural history of the disease.

Here, we analyzed data from the earliest cases detected in Antananarivo, Madagascar, and their intra domiciliary contacts to characterize epidemiological parameters of COVID‐19 during the first wave that affected the capital city of Madagascar. Using data from contact tracing, we evaluated SARS‐CoV‐2 transmission by estimating the serial interval, the household secondary attack rate (SAR), and the average number of family members infected by each index case. Furthermore, we describe risk factors for transmission and infection.

## 2 METHODS

We used an adaptation of generic protocols already in place in some countries, such as *“The First Few Hundred (FF100)” enhanced case and contact protocol* for pandemic influenza in the United Kingdom of Great Britain and Northern Ireland (11).

1. Case identification

On March 12–20, 2020, the Malagasy Ministry of Public Health tested for SARS‐CoV‐2 all travelers coming from Europe and China on international flights. Nasopharyngeal and oropharyngeal specimens (NP/OP) were sent to the virology unit at the Institut Pasteur de Madagascar, where they were tested for SARS‐CoV‐2 using real‐time RT‐PCR (RT‐qPCR) as previously described (12).

The population included in this study was among the first confirmed cases of COVID‐19.

A confirmed case is a person with laboratory confirmation of SARS‐CoV‐2 infection (RT‐qPCR), irrespective of clinical signs and symptoms. The index case (also called the primary case or infector) was identified through the national surveillance system. The index case is defined as the first individual of a household that tested positive for SARS‐CoV‐2 using RT‐qPCR. An imported case refers to a case with a history of travel from an affected area (Europe, America, African countries, Indian ocean region, and Asia) in the 14 days before the collection date of the first positive test result.

Close contacts were identified through contact tracing of a confirmed case and were defined as those who lived in the same house of a symptomatic index case up to 4 days before symptom onset or of an asymptomatic index case up to 4 days prior to the collection date of the first positive test result. The second case (infected) refers to a contact who tested positive using rRT‐PCR or that seroconverted according to an enzyme‐linked immunosorbent assay (ELISA). Seroconversion was defined as a serum specimen that tested negative at inclusion and became positive by either of the following tests during the follow‐up (supporting information Figure S1). The serological tests used during our study were a semiquantitative indirect ELISA for the detection of immunoglobulin G (IgG) to SARS‐CoV‐2 (ID Screen SARS‐CoV‐2‐N IgG Indirect ELISA Kit, ID.vet, Grabels, France) and a qualitative ELISA for the detection of total antibodies (including IgM and IgG) to SARS‐CoV‐2 (WANTAI SARS‐CoV‐2 Ab ELISA, Beijing Wantai Biological Pharmacy Enterprise Co., Beijing, China) provided by the WHO.

At the beginning of the outbreak on March 20 2020, confirmed cases were isolated and treated at one of the three main hospitals of Antananarivo (Befelatanana Hospital, Anosiala Hospital, and Andohatapenaka Hospital), regardless of the presence of symptoms.

NP/OP and blood specimens were collected from laboratory‐confirmed cases and household contacts as soon as possible after laboratory confirmation. For all laboratory‐confirmed index cases and household contacts, data were collected during the first visit and every 7 days until 21 days. NP/OP specimens were tested using RT‐qPCR within 24 h following collection, while sera were tested retrospectively at the end of the study.

- Epidemiological parameters

- *The incubation period* refers to the delay between exposure or contact with confirmed cases and symptom onset. We determined the left and right boundaries of the possible exposure and symptom onset times. Imported cases were assumed to have been exposed within 14 days prior to symptom onset. Cases without recent international travel history but with exposure to a confirmed case were assumed to be exposed from the time of earliest to latest possible contact with the case. Only cases for which it is possible to identify the earliest and latest time of exposure and who had a date of symptom onset were included in the estimation of the incubation period. The earliest exposure time was assumed to be within 15 days, and the latest exposure time was assumed to be within 30 days. Its distribution was calculated by fitting a parametric distribution (normal, gamma, lognormal, Weibull).
- Transmission was analyzed by examining the relationship between index cases and their close contacts.
- *The serial interval* is the average time expressed in days between the time of symptom onset of a primary case and that of a secondary case (13). Only pairs of symptomatic primary and secondary cases are included in the estimation of this indicator.
- The *household SAR* is calculated by dividing the household contacts who were later confirmed to have SARS‐CoV‐2 infection by the total number of household contacts included in the study.
- The *distribution of the average number of family members infected by each index case* was calculated from the number of secondary infections observed among close contacts of each index case.

All distributions of those parameters were also calculated by fitting parametric distributions (Normal, Lognormal, Gamma, Weibull) based on infector‐infected paired data.

‐ SAR was estimated for a range of factors using univariate analysis and multivariate mixed effects logistic regression models with a random intercept for households. Households with one or more household members were included. The following potential explanatory variables were examined: characteristics of the index case, including age, gender, comorbidities, and whether the case was symptomatic; characteristics of the contact, including gender and age group; and household size. A stepwise backward selection variable (less than 0.20) was used in univariate analyses to choose the final model in the multivariate analysis.

All analyses were conducted with R software (14).

- Ethical statement

Written informed consent was obtained from participants before enrolment in this study. It was approved by the Ethics Committee of Biomedical Research of the Ministry of Public Health of Madagascar (no. 058/MSANP/SG/AGMED/CERBM, March 30, 2020). For children and minors, written informed consent was obtained from parents or guardians on behalf of the minors enrolled in the study.

## 3 RESULTS

### 3.1 Characteristics of index cases

**FIGURE 1:**
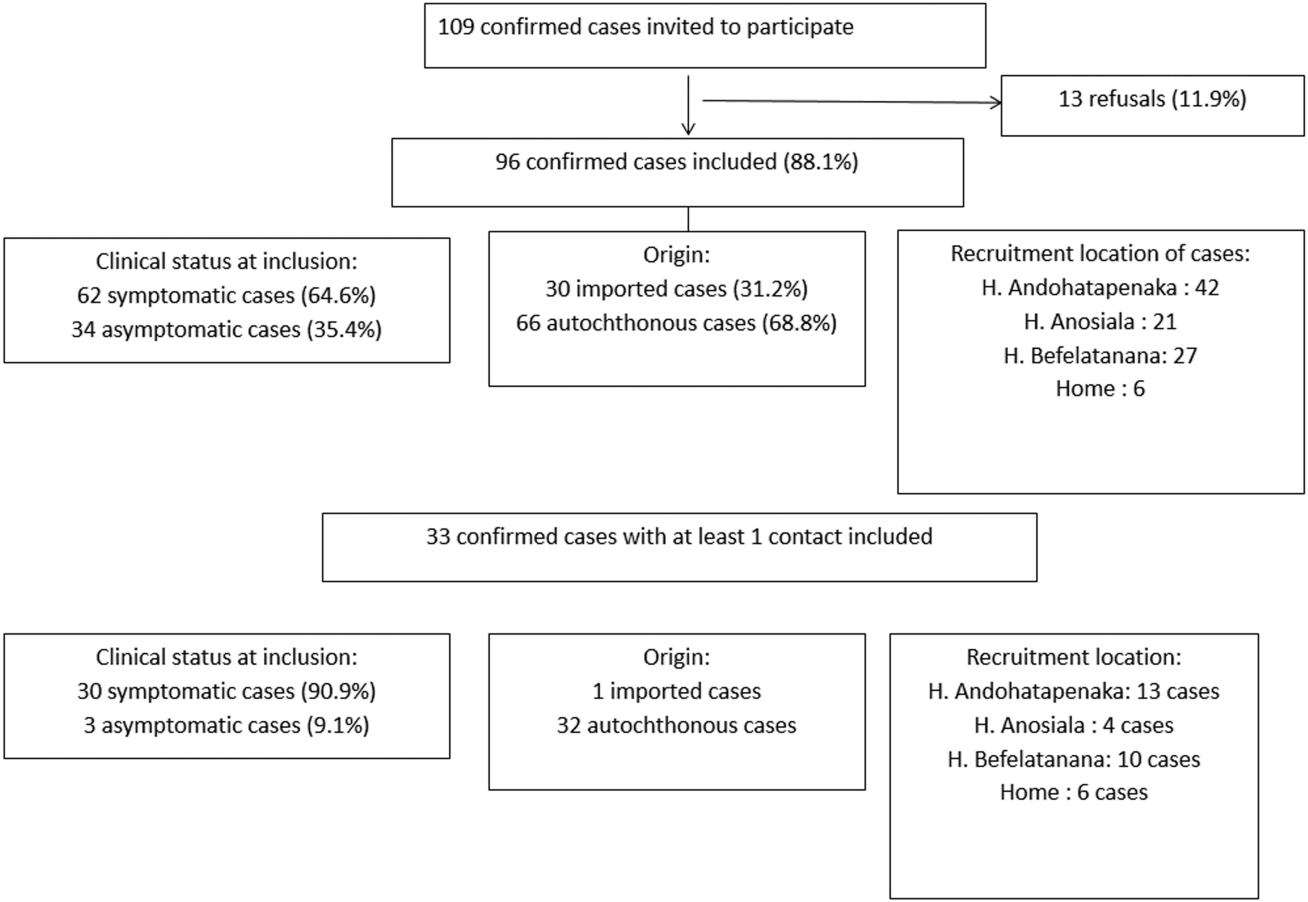
Flow chart of index cases included in the study according to their clinical status at inclusion, origin, and recruitment location

From March 19 to July 30, 2020, among the 109 confirmed cases invited to participate in our study, 96 were included, of which 30 were imported and 62 were symptomatic (Figure 1). The median age was 44 years (interquartile range IQR [30.4–58.9]). Males and females were equal in number (*n* = 48).

Most cases were hospitalized, but only seven cases presented complications such as acute respiratory distress syndrome that required admission to the intensive care unit. Among the 62 symptomatic cases, the main symptoms were cough (37.5%), fatigue (36.5%), fever (30.2%), muscle aches (22.9%), and runny nose (20.8%).

At inclusion, 91 (94.8%) index cases were sampled for serological analysis, of which 67% had already antibodies (IgG or total antibodies) to SARS‐CoV‐2 (seropositive).

Of the 96 index cases, 33 had at least one household contact who participated in the study. Among these 33 index cases, 30 were symptomatic (Figure 1). The median age was 42.8 years (IQR [31.1–52.2]), and there were 15 females and 18 males. At inclusion, 19 (57.5%) had positive serology on one of the two ELISA tests.

### 3.2 Characteristics of household contacts

**FIGURE 2:**
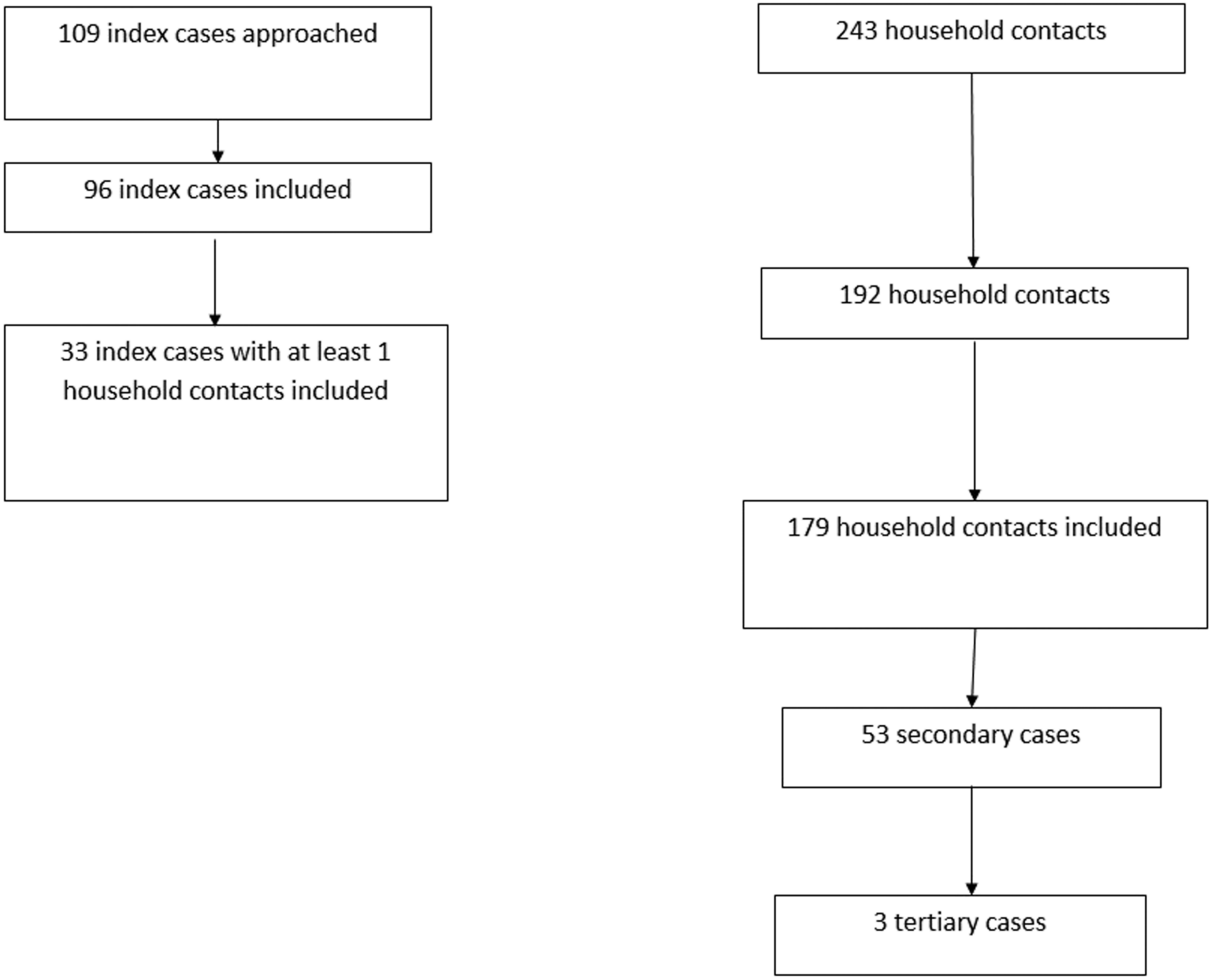
Flow chart of index cases and their close contacts that were included in the study

Overall, 192 household contacts from 33 index cases were invited to participate, of which 179 agreed to participate in the study (Figure 2) and were followed up for up to 21 days. The median age was 29.3 years (IQR = [15.6–53.5]). There were more females (54.2%) than males.

Among contacts, 73 (40.8%) were symptomatic at inclusion. The most common symptoms were runny nose (22.3%), cough (21.2%), headache (8.9%), fatigue (8.4%), and sore throat (7.8%).

Among the 179 contacts with complete follow‐up, 56 acquired infection with SARS‐CoV‐2 (Figure 2), among which three were tertiary cases. At the time of inclusion, among 41 contacts who tested positive by RT‐qPCR, 31.7% (13/41) were asymptomatic, and among those with antibodies to SARS‐CoV‐2 (supporting information Figure S1), 21.6% (8/37) had no symptoms. In total, 44.6% (25/56) of positive contacts were asymptomatic.

### 3.3 Epidemiological parameters

Incubation period, serial interval and average number of family members infected by each index case

The incubation period was calculated from 24 cases and 8 contacts for whom the earliest and latest time of exposure did not exceed 15 to 30 days. The empirical incubation period that fits the normal distribution was 4.1 days (95% CI [0.7–7.5]). The serial interval was 6.0 days (95% CI [2.4–9.6]) after analyzing the best‐fit Weibull distribution to 25 infector‐infected paired data (Table 1).

**TABLE 1:** Means, standard deviations, 95% confidence intervals, and medians of epidemiological parameters with all distributions analyzed and their Bayesian information criterion (BIC), Akaike information criterion (AIC), and log likelihood (LogLK)

Indicators	N	Distribution	BIC	AIC	LogLK	Mean (95%CI)	SD	Median (IQR)
Incubation period	32 (24 cases and 8 contacts)	Normal	240.8	237.8	−116.9	4.1 (0.7–7.5)	9.5	
		Lognormal	255.9	252.9	−124.5			
		Weibull	241.3	238.4	−117.2			
		Gamma	245.5	242.6	−119.3			
Serial interval	25 paired‐data	Normal	184.7	182.3	−89.1			
		Lognormal	185.6	183.1	−89.6			
		Weibull	179.3	176.9	−86.4	6.0 (2.4–9.6)	8.7	4.4 (−0.6–10.9)
		Gamma	180.2	177.7	−86.9			
Average number of family members infected by each index case	33 cases and 53 contacts					1.6 (0.9–2.3)	1.9	

Abbreviations: IQR, interquartile range; SD, standard‐deviation.

With 33 index cases and 53 positive household contact paired data, the average number of family members infected by each index case was estimated at 1.6 (95% CI [0.9–2.3]); when considering only a period of infectivity of 7 days, it was 1.7 (95% CI [0.9–2.6]).

### 3.4 Household transmission dynamics

The empirical SAR among close contacts was 29.6%, and after gamma distribution adjustment, the SAR was 38.8% (95% CI [19.5–58.2]). Unadjusted SAR odds ratios and multivariate analysis of secondary and tertiary cases by a range of explanatory variables are shown in Table 2. In both the univariate and multivariate analyses, there was a relationship between the age of contacts and SAR, with the highest SAR in contacts aged 35 years old or more. In the univariate analysis, there were neither significant effects of gender nor the presence of comorbidities in either the primary case or contacts nor the presence of symptoms in the index case. No significant effect of household size was found. Moreover, transmission seemed more likely to occur between index cases with comorbidities and contacts with comorbidities; however, it was not significant in the multivariate analysis.

**TABLE 2:** Unadjusted secondary attack rates, odds ratio for secondary infection, and multivariate analysis

	Univariate analysis					Final model	
	Variable levels	SAR (%)	SAR95%CI	Crude OR	*p*	Adjusted OR	*p*
**Household size**	< 4 persons	37.9	21.3–57.6	1			
	4–8 persons	44.9	30.9–59.6	1.4 (0.1–15.6)	0.7		
	≥ 9 persons	21.3	13.7–31.2	0.4 (0.03–3.7)	0.3		
**Interactions**	**Sex**						
	Male ‐ > Male	29.7	16.4–47.2	1			
	Male ‐ > Female	39.6	26.7–53.9	1.7 (0.5–6.3)	0.4		
	Female ‐ > Male	25.0	13.7–40.6	0.8 (0.1–7.5)	0.8		
	Female ‐ > Female	26.3	13.9–43.4	0.7 (0.1–7.3)	0.7		
	**Comorbidities**						
	None‐ > none	25.9	16.9–37.4	1			
	None ‐ > comorbidities	19.2	7.3–39.9	0.5 (0.1–1.9)	0.3		
	Comorbidities ‐ > none	39.1	25.4–54.6	3.9 (0.5–54.9)	0.2		
	Comorbidities ‐ > comorbidities	43.5	23.9–65.1	15.8 (1.6–366.1)	0.03		
**Characteristics of contact**	**Sex**						
	Male	27.7	18.1–38.4				
	Female	34.1	24.6–44.8	1.3 (0.5–3.3)	0.5		
	**Age (years)**						
	<15	25.0	13.2–41.5	1		1	
	15–34	24.6	14.5–38.0	1.2 (0.3–4.8)	0.8	1.2(0.3–4.8)	0.8
	35–54	50.0	33.6–66.4	6.5 (1.6–31.3)	0.01	6.5 (1.6–31.3)	0.01
	55 et+	30.2	17.6–46.3	3.3 (0.8–16.7)	0.1	3.3 (0.8–16.7)	0.1
**Characteristics of primary case**	**Sex**						
	Male	35.6	25.9–46.4	1			
	Female	25.6	16.9–36.6	0.5 (0.1–4.1)	0.5		
	**Age (years)**						
	<25	16.7	2.9–49.1	1			
	25–49	27.6	19.8–36.8	3.0 (0.1–148.4)	0.5		
	≥ 50	43.2	28.6–58.8	14.5 (inf)	0.1		
	**Respiratory symptoms**						
	No	11.7	3.8–28.4				
	Yes	35.5	27.6–44.1	6.5 (inf)	0.1		

## 4 DISCUSSION

In the capital city of Antananarivo, we analyzed data from the earliest cases of COVID‐19 and their intradomiciliary contacts to characterize key aspects of COVID‐19 transmission during the first wave of SARS‐CoV‐2. We included 96 cases and 179 contacts. We estimated a high SAR of 38.8% (95% CI [19.5–58.2]) within households, a serial interval of 6.0 days (95% CI [2.4–9.6]) and an average number of family members infected by each index case of 1.6 (95% CI [0.9–2.3]). We found that SAR was higher when contacts were older than 35 years old (50%). The mean incubation period was 4.1 (CI95% [0.7–7.5]).

Our estimated SAR in Antananarivo was higher than those reported in Asia, mainly in Singapore (15) and China (16, 17) and was similar to the household SAR found in the United Kingdom (18) and in some US states, such as Tennessee and Wisconsin (19). The heterogeneity in SAR across different regions might be explained by differences in control measures and crowdedness in households (17). This high SAR we reported in the current study reflects the existence of high transmission within households. In Madagascar, at the beginning of the outbreak, all people confirmed to have SARS‐CoV‐2 infection were isolated in hospitals regardless of symptoms. Subsequently, in June 2020, the national recommendation changed, and infected individuals with asymptomatic or mild symptoms were treated and isolated at home with their household contacts for 14 days. During the quarantine, many measures were recommended, including mask‐wearing at home in shared spaces and physical separation from the infected person (separate room) when feasible. However, in low‐income countries such as Madagascar, this type of recommendation might be difficult to achieve. Overcrowding is common at the household level, and houses are generally poorly ventilated. The national average size of households is 4.2; in urban areas, it is 4 (20), and at the national level, Malagasy households occupy an average surface area of 26 m^2^ (21). Little is known about the household transmission indicators of COVID‐19 in low‐income countries where health system resources are weak and limited.

Our results suggested a higher transmission among household contacts aged 35 years and older compared to children. A systematic review and meta‐analysis by Madewell ZJ et al. (22) reported that the SAR of SARS‐CoV‐2 to adults was higher than that to children, assuming that adults might be more susceptible to SARS‐CoV‐2 than children when they exposed themselves to the same sources of infection. Data collected in Madagascar from March to September 2020 confirmed the same finding and suggested that individuals aged 50 years and older had a higher probability of having a positive RT‐qPCR for SARS‐CoV‐2 (12).

We found an incubation period of 4.1 days (95% CI [0.7–7.5]), which is similar to those reported elsewhere (3, 23). This estimate provides evidence to support a 14‐day period of quarantine for infected and exposed persons. We reported a wider serial interval, which is similar to that found in China (3, 24). Two hypotheses could explain these data: the memorization bias of the date of symptom onset and intrahousehold contamination from asymptomatic cases due to the lack of respect for protective/distancing measures. Indeed, we cannot exclude potential transmission from asymptomatic cases since asymptomatic infections among index cases and secondary cases were 35.4% and 44.6%, respectively (25/56). In addition, at the time of inclusion, (13/41) 31.7% of contacts tested positive for the virus (RT‐qPCR), and (8/37) 21.6% with antibodies to SARS‐CoV‐2 had no symptoms. These results may imply that they had been infected before the index cases; in fact, the initial household member who had been diagnosed as infected with SARS‐CoV‐2 was considered the index patient, but we could not confirm that other household members were infected concurrently but developed symptoms at different times or remained asymptomatic. Onward transmission has been documented from asymptomatic SARS‐CoV‐2 cases, especially in the household setting (25, 26), even if data are still scarce.

Our study had several limitations. Even if living in the same household might increase the risk of acquiring infection, we cannot exclude infections from outside of the home leading to higher apparent secondary infection rates. Second, the median time between the index case rRT‐PCR confirmation and the date of inclusion in the study (12 days) could have impacted the biological results during enrolment (some household members were already infected at enrolment) and the estimation of transmission indicators. However, we found that the transmission indicators did not differ according to rRT‐PCR confirmation and the inclusion date of the study. Third, we included 31.2% imported cases in the study that did not have household contacts, which reduced the sample size on which we could calculate transmission indicators and might explain the wide confidence interval in some analyses. Finally, we conducted early analyses of the COVID‐19 pandemic among cases and household contacts which may lead to selection bias and biases in the estimation of the incubation period. As the inclusion in the study was limited to the early phase of the outbreak when the epidemic was rapidly growing, we might have missed other infected people with longer incubation periods.

Our results confirm that the household is an important venue for transmission and could explain the intensity and rapid spread of the virus during the second wave that started in early March 2021. To avoid transmission in the community, control measures such as appropriate isolation of cases and their household contacts should be adopted.

## AUTHOR CONTRIBUTIONS

**Rila Ratovoson:** Formal analysis; supervision. **Rado Razafimahatratra:** Investigation; supervision. **Lova Randriamanantsoa:** Investigation; supervision. **Mihaja Raberahona:** Investigation; supervision. **Hasina Joelinotahiana Rabarison:** Investigation. **Fara Rahaingovahoaka:** Formal analysis. **Emmanuel Harizaka Andriamasy:** Investigation; supervision. **Perlinot Herindrainy:** Methodology; supervision. **Norosoa Razanajatovo:** Supervision. **Soa Fy Andriamandimby:** Supervision. **Philippe Dussart:** Methodology; supervision; validation. **Matthieu Schoenhals:** Methodology; supervision; validation. **Mamy Jean de Dieu Randria:** Investigation; supervision; validation. **Jean Michel Heraud:** Conceptualization; funding acquisition; methodology; supervision; validation.

## CONFLICT OF INTEREST

The authors declare no competing interests.

### PEER REVIEW

The peer review history for this article is available at https://publons.com/publon/10.1111/irv.12896.

## Supporting information


**Supporting Information Figure S1.** Flowchart used for identification of secondary and tertiary cases during the follow‐up of close contacts in the statistical analysis

## Data Availability

The data that support the findings of this study are available from the corresponding author upon reasonable request.

## References

[1] World Health Organization . Statement on the second meeting of the International Health Regulations (2005) Emergency Committee regarding the outbreak of novel coronavirus (2019‐nCoV): WHO; 2020 [cited 2020 30‐10‐2020].

[2] Lai C , Shih T , Ko W , Tang H , Hsueh P . Severe acute respiratory syndrome coronavirus 2 (SARS‐CoV‐2) and coronavirus disease‐2019 (COVID‐19): The epidemic and the challenges. Int J Antimicrob Agents. 2020;55(3):105924. Pubmed Central PMCID: PMC7127800 32081636 10.1016/j.ijantimicag.2020.105924 PMC7127800

[3] Li Q , Guan X , Wu P , Wang X , Zhou L , Tong Y . Early transmission dynamics in Wuhan, China, of novel coronavirus‐infected pneumonia. N Engl J Med. 2020;382(13):1199‐1207. 31995857 10.1056/NEJMoa2001316 PMC7121484

[4] Docherty A , Harrison E , Green H , Hardwick H . Features of 20 133 UK patients in hospital with covid‐19 using the ISARIC WHO clinical characterisation protocol: Prospective observational cohort study. BMJ. 2020;369:m1985. 32444460 10.1136/bmj.m1985 PMC7243036

[5] Grasselli G , Zangrillo A , Zanella A , Antonelli M , Cabrini L . Baseline characteristics and outcomes of 1591 patients infected with SARS‐CoV‐2 admitted to ICUs of the Lombardy region. Italy JAMA. 2020;323(16):1574‐1581. 32250385 10.1001/jama.2020.5394 PMC7136855

[6] Petrilli C , Jones S , Yang J , Rajagopalan H , O'Donnell L , Chernyak Y . Factors associated with hospital admission and critical illness among 5279 people with coronavirus disease 2019 in New York City: Prospective cohort study. BMJ. 2020;369:m1966. 32444366 10.1136/bmj.m1966 PMC7243801

[7] Laxminarayan R , Wahl B , Dudala S , Gopal K , Mohan C , Neelima S . Epidemiology and transmission dynamics of COVID‐19 in two Indian States. Science. 2020;370(6517):691‐697. 33154136 10.1126/science.abd7672 PMC7857399

[8] Rakotosamimanana N , Randrianirina F , Randremanana R . GeneXpert for the diagnosis of COVID‐19in LMICs. Lancet Glob Health. 2020;8(12):e1457‐e1458. 33091372 10.1016/S2214-109X(20)30428-9 PMC7572106

[9] Rosenthal PJ , Breman JG , Djimde AA , et al. COVID‐19: Shining the Light on Africa. Am J Trop Med Hyg. 2020;102(6):1145‐1148. PubMed PMID: 32372749. eng 32372749 10.4269/ajtmh.20-0380 PMC7253089

[10] WHO . WHO Coronavirus Disease (COVID‐19) Dashboard: WHO Health Emergency Dashboard; 2020 [cited 2020 04/11/2020].

[11] Public Health England . "The First Few Hundred (FF100)" Enhanced Case and Contact Protocol v12 United Kingdom: Public Health England. Available from: https://assets.publishing.service.gov.uk/government/uploads/system/uploads/attachment_data/file/360190/2012_13_FF100_Protocol_H7N9_ver_12.pdf

[12] Randremanana RV , Andriamandimby SF , Rakotondramanga JM , et al. The COVID‐19 epidemic in Madagascar: Clinical description and laboratory results of the first wave, March‐September 2020. Influenza Other Respir Viruses. 2021 Feb 15. eng 10.1111/irv.12845 PMC8013501 33586912

[13] Nishiura H , Linton N , Akhmetzhanov A . Serial interval of novel coronavirus (COVID‐19) infections. Int J Infect Dis. 2020;93:284‐286. 32145466 10.1016/j.ijid.2020.02.060 PMC7128842

[14] R Core Team. R: A language and environment for statistical computing. Vienna, Austria: R foundation for statistical computing; 2018.

[15] Ng OT , Marimuthu K , Koh V , et al. SARS‐CoV‐2 seroprevalence and transmission risk factors among high‐risk close contacts: A retrospective cohort study. The Lancet Infectious diseases. 2021;21(3):333‐343. PubMed PMID: 33152271. Epub 11/02. eng 33152271 10.1016/S1473-3099(20)30833-1 PMC7831879

[16] Li W , Zhang B , Lu J , et al. Characteristics of households transmission of COVID‐19. Clin Infect Dis. 2020 Nov 5;71(8):1943‐1946. 32301964 10.1093/cid/ciaa450 PMC7184465

[17] Li F , Li Y , Liu M , et al. Household transmission of SARS‐CoV‐2 and risk factors for susceptibility and infectivity in Wuhan: A retrospective observational study. Lancet Infect Dis. 2021 Jan 18;S1473‐3099(20):30981‐30986. 10.1016/S1473-3099(20)30981-6 PMC7833912 33476567

[18] Bernal J , Panagiotopoulos N , Byers C , et al. Transmission dynamics of COVID‐19 in household and community settings in the United Kingdom. medRxiv. 2020 August 22. 10.2807/1560-7917.ES.2022.27.15.2001551 PMC9012093 35426357

[19] Grijalva C , Rolfes MA , Zhu Y , et al. Transmission of SARS‐COV‐2 Infections in Households—Tennessee and Wisconsin, April–September 2020. MMWR Morb Mortal Wkly Rep. 2020 Nov 6;69(44):1631‐1634. eng 33151916 10.15585/mmwr.mm6944e1 PMC7643897

[20] Institut National des Statistiques . Recensement Général de la Population et de l'Habitation (RGPH‐3). Antanananarivo: INSTAT, 2020.

[21] Institut National des Statistiques . Enquête Périodique auprès des ménages—Rapport principal. INSTAT, 2010.

[22] Madewell ZJ , Yang Y , Longini IM Jr , Halloran ME , Dean NE . Household transmission of SARS‐CoV‐2: A systematic review and meta‐analysis. JAMA Netw Open. 2020 Dec 1;3(12):e2031756. eng 33315116 10.1001/jamanetworkopen.2020.31756 PMC7737089

[23] Tu H , Tu S , Gao S , Shao A , Sheng J . Current epidemiological and clinical features of COVID‐19; A global perspective from China. J Infect. 2020 Jul;81(1):1‐9. 32315723 10.1016/j.jinf.2020.04.011 PMC7166041

[24] Bi Q , Wu Y , Mei S , et al. Epidemiology and transmission of COVID‐19 in 391 cases and 1286 of their close contacts in Shenzhen, China: A retrospective cohort study. Lancet Infect Dis. 2020 Aug;20(8):911‐919. Epub 2020 Apr 27. eng 32353347 10.1016/S1473-3099(20)30287-5 PMC7185944

[25] Bai Y , Yao L , Wei T , et al. Presumed asymptomatic carrier transmission of COVID‐19. JAMA. 2020 Apr 14;323(14):1406‐1407. 32083643 10.1001/jama.2020.2565 PMC7042844

[26] Chau NVV , Thanh Lam V , Thanh Dung N , et al. The natural history and transmission potential of asymptomatic SARS‐CoV‐2 infection. Clin Infect Dis. 2020 Jun 4;71(10):2679‐2687. eng 32497212 10.1093/cid/ciaa711 PMC7314145

