# Supplementary material for: Household transmission of COVID‐19 among the earliest cases in Antananarivo, Madagascar
Source: Influenza Other Respir Viruses. 2021 Aug 10;16(1):48–55. doi: 10.1111/irv.12896 (PMC8447323; doi:10.1111/irv.12896)
Supplement: Supplementary file 1 — Supporting Information Figure S1. Flowchart used for identification of secondary and tertiary cases during the follow‐up of close contacts in the statistical analysis [file IRV-16-48-s001.docx]

**Supplementary files**

Close contacts included N=179

Inclusion
 (Day 1)

PCR

Serology

**Positive
= n1 = 41**

Positive^#^ (n=37)

Negative^#^

Negative

Follow-up (Day 7)

PCR
(n=113)

**Positive
= n2 = 6**

Negative

Follow-up (Day 14)

PCR
(n=107)

**Positive
= n3 = 4**

Negative

Follow-up (Day 21)

PCR
(n=103)

**Positive
= n4 = 3**

Negative

Serology

**Positive
= n5 = 2**

Negative

**^#^** Close contacts with positive serology and negative rRT-PCR at inclusion (n=25) were classified as already infected before their inclusion in the study.

**Secondary and tertiary cases = n1 + n2 + n3 + n4 + n5 = 56**

**Supplementary figure 1.** Flowchart used for identification of secondary and tertiary cases during the follow-up of close contacts in the statistical analysis
